# Bariatric Surgery Improves Renal Function in Patients With Obesity

**DOI:** 10.7759/cureus.17458

**Published:** 2021-08-26

**Authors:** Oaklee L Abernathy, Hayrettin Okut, Bobbie G Paull-Forney, Tiffany E Schwasinger-Schmidt

**Affiliations:** 1 Internal Medicine, Kansas University School of Medicine-Wichita, Wichita, USA; 2 Preventive Medicine, Kansas University School of Medicine-Wichita, Wichita, USA; 3 Weight Management Clinic, Ascension Via Christi Hospital, Wichita, USA

**Keywords:** community-based weight loss intervention, bariatric and endocrine surgery, impaired renal function, weight loss and obesity, lifestyle intervention, behavioral intervention, obesity, kidney function, weight loss, hypertension

## Abstract

Background

Obesity affects 93.3 million adults in the United States and is a predisposing factor for the development and progression of chronic kidney disease (CKD). The objective of this study is to examine the association between weight loss and renal function in participants undergoing bariatric surgery following a 12-week multidisciplinary, community-based weight loss program.

Methodology

This is a retrospective chart review of participants who voluntarily enrolled in a 12-week multidisciplinary weight loss program prior to bariatric surgery from 2009 to 2018. The primary outcome was to assess the association between weight loss and renal function in participants undergoing bariatric surgery. Secondary outcomes included changes in hemoglobin A1c, lipids, fasting glucose, and blood pressure.

Results

Among the 55 participants, baseline glomerular filtration rate (GFR) was 49 mL/min/m^2^, 80% were female, and the average baseline weight was 131 kg. At one-year post-intervention, 69% of patients improved in the CKD stage, with 45% of the participants improving from stage 3A to stage 2. GFR improved to 15 mL/min/1.73m^2^ (p = 0.025), and there was a negative correlation (r_s_ = -0.3556) between weight and GFR (p = 0.013). Participants with hyperlipidemia had a 12 mL/min/1.73m^2^ rise in GFR, while participants without the diagnosis at one year had a 24 mL/min/1.73m^2 ^rise in GFR (p = 0.007).

Conclusions

This study demonstrated improved renal function and reduced progression of CKD following a combined lifestyle and surgical intervention, indicating the importance of a comprehensive approach for the management of the chronic disease.

## Introduction

Obesity affects 93.3 million adults in the United States and current projections indicate that nearly half of the population aged 18 and older will be obese within the next 10 years [1]. Obesity is an independent risk factor for the development of multiple comorbid conditions including type II diabetes mellitus, hypertension, coronary artery disease, nonalcoholic fatty liver disease, and chronic kidney disease (CKD) [2]. The mechanisms linking obesity with cardiovascular disease have been well established; however, the direct effects of obesity on the kidney are not fully understood [3]. Given the significant contribution of renal function to vascular regulation and remodeling, the effects of obesity on the kidney may result in disproportionate negative effects on the cardiovascular system, thus increasing overall morbidity and mortality [4,5].

In patients with obesity, the renal system causes increased glomerular hyperfiltration to compensate for metabolic demands placed on the body, resulting in glomerulomegaly and focal glomerulosclerosis [6]. Renal hyperfiltration increases the intraglomerular pressure which can result in proteinuria and decreased glomerular filtration rate (GFR), putting patients at risk for progression of CKD and development of end-stage renal disease (ESRD) [7-9]. Patients with CKD have higher rates of hospitalizations and increased mortality compared to patients with normal renal function [10]. Numerous biological mechanisms contribute to the development of CKD in patients with obesity, including adiposity-induced inflammatory states, hyperfiltration, changes in renal blood flow, oxidative stress, renal sclerosis, and activation of the renin-angiotensin system [11,12].

Bariatric surgery is currently indicated for patients with a body mass index (BMI) greater than 40 kg/m^2^ or for patients with a BMI greater than or equal to 35 kg/m^2^ who also have at least one obesity-related comorbid condition, including type II diabetes, hypertension, or cardiovascular disease [13]. Bariatric surgery has been associated with improved glycemic control and increased rates of diabetes remission up to five years post-surgical intervention, with 51% of patients stopping diabetic medications by one year [14]. Additionally, patients with post-surgical diabetes tended to have higher pre-surgical hemoglobin A1c (HbA1c) levels and reduced estimated body weight loss following surgery. Bariatric surgery has decreased the number of antihypertensive, diabetic, and lipid-lowering medications used three years post-intervention [15]. In patients who were followed for 14 years, bariatric surgery was associated with a two-thirds reduction in the first-time incidence of myocardial infarction, stroke, and heart failure [16].

Bariatric surgery has been associated with a 58% reduction in the risk of proteinuria and albuminuria and a 54% reduction in the risk of hyperfiltration in patients with obesity and impaired renal function [12]. Additionally, bariatric surgery is associated with improvements in diabetes and hypertension which are risk factors for the development of CKD. However, few studies analyze the long-term effects of bariatric surgery on renal-associated morbidity.

This study aimed to take a real-world approach to chronic care management by focusing on patient-controlled factors such as lifestyle modifications. Studies have shown improved post-surgical outcomes if lifestyle and behavioral changes are incorporated prior to surgery. This study sought to examine the association between weight loss following bariatric surgery and renal function among patients who participated in a 12-week intensive lifestyle modification weight loss program prior to surgery.

## Materials and methods

Research participants

**Figure 1:**
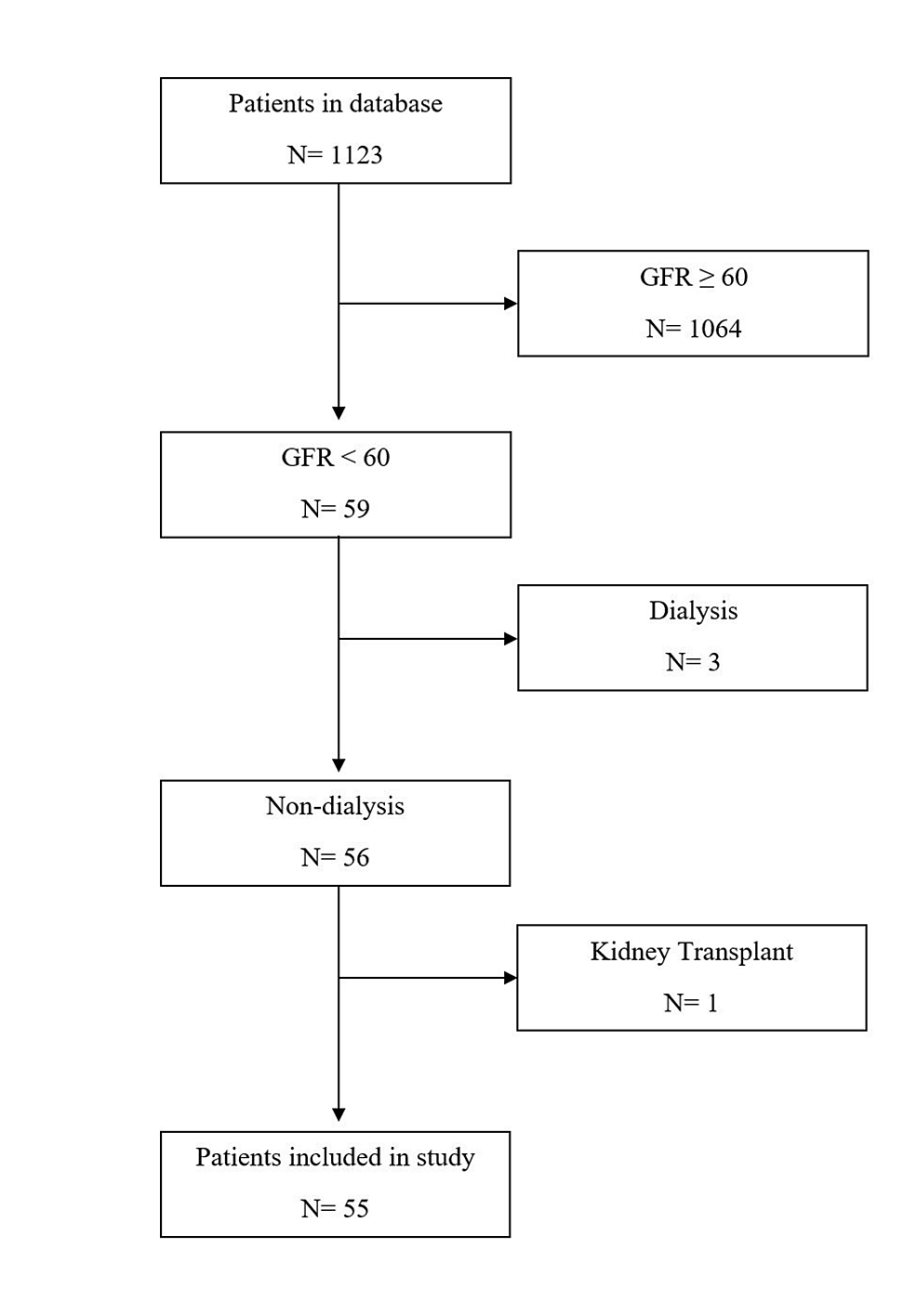
Flowchart of patients who met the inclusion and exclusion criteria for the study sample. GFR: glomerular filtration rate

Participants 18 years or older voluntarily enrolled in a 12-week multimodal weight loss program through a weight management clinic in the Midwest from January 1, 2009 through December 31, 2018, prior to completing bariatric surgery. Patients who were on dialysis, had a previous kidney transplant, or had a GFR greater than or equal to 60 mL/min/m^2^ were excluded from the study (Figure 1). All participants met the Health Management Resources (HMR) Program Medical Guidelines, which excluded patients with renal failure, eating disorders, severe liver disease, Cushing’s syndrome, active malignancy, osteomyelitis, bacterial endocarditis, tuberculosis, pregnant women, or substance abusers. Individuals who did not follow up at one year (±six months) after bariatric surgery were not included in the study.

Preoperative lifestyle-modification program

Prior to referral for bariatric surgery, participants were required to successfully complete a 12-week multidisciplinary preoperative program focused on healthy lifestyle interventions. Participants consumed meal replacements (entrees, soups, cereals, and shakes) and five servings of fruits and vegetables daily with a minimum intake of 800 kilocalories per day. Participants were additionally encouraged to complete 300 kilocalories per day of physical activity.

Participants met weekly with the staff to report compliance with established intake goals. They attended group classes that provided a sense of community and assisted with accountability and support throughout the 12-week program. Comorbid conditions were optimized by a physician through medical and lifestyle management.

Outcome measurement and data collection

The primary outcome of this study was the association between weight loss and GFR. GFR was compared at baseline, following completion of the 12-week preoperative program, and following bariatric surgery at three months, six months, and one year (±six months). GFR was calculated utilizing the CKD-EPI formula from the National Kidney Foundation (GFR = 141 × min (Scr/κ,1)^α^ × max(Scr/κ,1)^-1.209^ × 0.993^Age^ × 1.018 [if female] × 1.159 [if black]) [17]. The CKD stage of each patient was determined using the GFR calculation. A GFR of ≥90 mL/min/1.73m^2^ is classified as stage 1 CKD, GFR of 60-89 mL/min/1.73m^2^ is stage 2, GFR of 45-59 mL/min/1.73m^2^ is stage 3A, GFR of 30-44 mL/min/1.73m^2^ is stage 3B, GFR of 15-29 mL/min/1.73m^2^ is stage 4, and a GFR of <15 mL/min/1.73m^2^ is stage 5 CKD.

Secondary outcomes included fasting plasma glucose, HbA1c, total cholesterol, triglycerides, high-density lipoproteins (HDL), low-density lipoproteins (LDL), and systolic and diastolic blood pressure. Patients with hypertension were defined as systolic blood pressure greater than 130 mmHg, diastolic blood pressure greater than 80 mmHg, or those taking antihypertensive medications. Patients with diabetes were defined as those having a fasting plasma glucose greater than or equal to 125 mg/dL, HbA1c greater than 6.5%, or taking medications for diabetes. Patients with hyperlipidemia were defined as those having an HDL less than or equal to 40 mg/dL, triglycerides greater than or equal to 150 mg/dL, LDL greater than or equal to 140 mg/dL, or those taking cholesterol-lowering medications.

Study data were managed using a secure Research Electronic Data Capture (REDCap) tool [18]. This study was approved by a local Human Subjects Committee and Institutional Review Board. The staff at the weight management clinic provided investigators with a clinical dataset consisting of patients who completed the preoperative lifestyle-modification program and received bariatric surgery at a Midwestern weight loss clinic from January 1, 2009 to December 31, 2018. Only participants who met the inclusion and exclusion criteria were enrolled in the study.

Statistical analysis

Data were analyzed using SAS version 9.4 (2019, SAS Int. Inc., Cary, NC). Frequencies, proportions, means, and standard deviations were generated from the initial dataset. Likelihood ratio chi-square and Fisher’s exact tests were used to test for associations between two nominal or categorical variables. Prior to the main analyses, the Shapiro-Wilk test was conducted to test for the normal distribution of the outcomes. The relationship between weight loss and GFR was evaluated using the Spearman Rho correlation coefficient. The main analyses were conducted through a random coefficient model under the framework of a generalized linear mixed model. This approach accounted for the individual fluctuations during the measurement time points, addressed questions of scientific interest about trajectories for individual units either in the study or future units, and accounted for within-individual and among-individual variation. The model parameter estimates were obtained, including maximum likelihood, with adaptive quadrature. Hannan-Quinn information criterion was considered in the model to select the correct set of variance and covariance patterns that better fit the outcome variable. The Wilcoxon signed-rank test was conducted to test the difference between baseline and final measurements of GFR based on hypertension, hyperlipidemia, and diabetes diagnosis. All statistical tests at p ≤ 0.05 were noted as significant.

## Results

A total of 55 participants were enrolled in the study, with the majority being female (n = 44; 80%) and Caucasian (n = 44; 80%) with a mean age of 59 ± 10 years (range: 38 to 77 years) (Table 1). Approximately 84% of the participants were classified as class III obesity (n = 46), while the remaining 16% were classified as class II obesity (n = 9). At baseline, 91% of participants were diagnosed with hypertension, 60% were diagnosed with diabetes mellitus, and 78% were diagnosed with hyperlipidemia (Table 2).

**Table 1:** Demographics of bariatric surgery patients with a GFR of <60 mL/min/m2 who participated in an eight-week multidisciplinary weight loss program. GFR: glomerular filtration rate

		Frequency	Percentage
Age (Years)			
	30-39	3	5
	40-49	8	15
	50-59	12	22
	60-69	22	40
	70-79	10	18
Gender			
	Female	44	80
	Male	11	20
Race			
	American Indian	1	2
	African American	6	11
	Caucasian	44	80
	Other	3	5
	Not reported	1	2
Ethnicity			
	Hispanic or Latino	2	4
	Not Hispanic or Latino	52	94
	Not reported	1	2
Weight (kg)			
	<90	1	2
	90-109	12	22
	110-129	16	29
	130-149	11	20
	150-170	9	16
	>170	6	11

**Table 2:** Laboratory values and clinical diagnoses at baseline, prior to surgery, three months, six months, and one year after the surgical intervention in patients who participated in a 12-week multidisciplinary weight loss program prior to bariatric surgery. * P-value is in reference to the difference between one year and baseline. GFR: glomerular filtration rate; HDL: high-density lipoprotein; LDL: low-density lipoprotein

Laboratory values	Baseline (n)	Prior to surgery (n)	3 Months (n)	6 Months (n)	1 Year (n)	P-value*
	Laboratory values below are presented as mean ± standard deviation (frequency)					
GFR	49 ± 8 (55)	54 ± 13 (55)	62 ± 14 (50)	63 ± 13 (41)	64 ± 15 (51)	0.0253
Weight (kg)	131 ± 26 (55)	N/A	N/A	N/A	98 ± 30 (50)	0.0001
Creatinine	1.3 ± 0.4 (55)	1.3 ± 0.3 (55)	1.1 ± 0.3 (50)	1.1 ± 0.2 (41)	1.1 ± 0.3 (51)	0.0001
Total cholesterol	188 ± 59 (54)	N/A	181 ± 52 (23)	165 ± 46 (20)	169 ± 47 (29)	0.3756
Triglycerides	185 ± 140 (54)	N/A	158 ± 101 (23)	125 ± 87 (20)	137 ± 81 (29)	0.0253
HDL	48 ± 16 (54)	N/A	45 ± 11 (23)	52 ± 16 (20)	55 ± 33 (28)	0.1042
LDL	106 ± 51 (52)	N/A	106 ± 38 (21)	93 ± 28 (20)	94 ± 39 (26)	0.8057
Fasting glucose	140 ± 71 (55)	144 ± 84 (55)	117 ± 58 (50)	107 ± 44 (41)	114 ± 43 (50)	0.0196
Hemoglobin A1c	6.9 ± 1.5 (52)	7.3 ± 1.6 (13)	6.7 ± 1.4 (18)	6.5 ± 1.5 (17)	6.7 ± 1.3 (16)	0.0136
Systolic blood pressure	136 ± 20 (54)	N/A	N/A	N/A	133 ± 21 (46)	0.1959
Diastolic blood pressure	76 ± 14 (54)	N/A	N/A	N/A	77 ± 13 (46)	0.9265
Clinical diagnosis	Baseline % (n)	Prior to surgery % (n)	3 Months % (n)	6 Months % (n)	1 Year % (n)	P-value*
	Clinical diagnosis values below are presented as percentage (frequency)					
Hypertension	91 (50)	N/A	N/A	N/A	85 (39)	0.4161
Diabetes	60 (33)	N/A	N/A	N/A	36 (19)	0.0001
Hyperlipidemia	78 (43)	N/A	N/A	N/A	55 (28)	0.0860

The average weight at baseline was 131 ± 26 kg and decreased to 98 ± 30 kg at one year, representing an average of 25% total body weight loss at one-year post-surgical intervention. The average GFR at baseline was 49 ± 8 mL/min/m^2^ and continued to increase at three months (62 ± 14 mL/min/m^2^), six months (63 ± 13 mL/min/m^2^), and one year (64 ± 15 ml/min/m^2^) (Table 2). Creatinine decreased from 1.3 mg/dL at baseline to 1.1 mg/dL at one year (p < 0.001). At baseline, 80% (n = 44) of the patients had CKD stage 3A and 16% (n = 9) had CKD stage 3B. At one year, 47% (n = 24) of patients had CKD stage 2 and 41% (n = 24) had CKD stage 3A, representing a 69% improvement in CKD stage at one-year post-surgical intervention (Table 3). GFR improved on average by 31% at one year following surgery (p = 0.025). Additionally, there was a negative correlation (r_s_ = -0.3556, p = 0.013) between weight loss and GFR.

**Table 3:** CKD stage at baseline and one year after bariatric surgery in patients who participated in a 12-week multidisciplinary weight loss program. CKD: chronic kidney disease

		Frequency	Percentage
Baseline CKD stage			
	3A	44	80
	3B	9	16
	4	2	4
One-year CKD stage			
	1	4	8
	2	24	47
	3A	21	41
	3B	2	4

Secondary outcome measures, including triglycerides, hbA1C, and fasting glucose, showed a significant improvement from baseline to one-year post-surgical intervention (Table 2). Triglycerides decreased from 185 mg/dL at baseline to 137 mg/dL at one-year post-surgical intervention, representing a 26% reduction. HbA1c decreased from 6.9% at baseline to 6.7% at one year, representing a 3% decrease. Fasting glucose decreased from 140 mg/dL at baseline to 114 mg/dL at one year, representing a 19% decrease.

Patients with hyperlipidemia had a smaller increase in GFR from baseline to one year when compared to patients without hyperlipidemia (χ^2^ (2, N = 51) = 6.52; p = 0.007)). There was no statistically significant difference in GFR change from baseline to one-year post-intervention in patients with diabetes or hypertension compared to patients without the diagnoses.

## Discussion

This study confirms that significant improvements in renal function in patients with obesity are achievable through community-based weight loss programs that combine lifestyle and surgical interventions. A strong positive correlation was noted with weight loss and improvements in renal function with additional improvements noted in triglycerides, HbA1c, and fasting plasma glucose. These reductions in cardiovascular risk factors are likely to have a significant impact on overall morbidity and mortality leading to improved overall cardiovascular outcomes in patients with obesity given that cardiovascular disease disproportionately affects this population [19].

On average, participants in the study lost 25% of the total body weight at one-year post-surgical intervention with a 31% improvement in GFR. Additionally, 69% of participants improved in the CKD stage. Previous studies support this finding with a 9.84 mL/min/1.73 m^2^ improvement in GFR at three-year post-surgical intervention [20]. Additional studies in patients with a baseline GFR between 45 and 59 mL/min/1.73 m^2^ showed improved CKD risk by 78% at one year and 56% at seven years after surgical intervention [21]. While the findings support the current literature, it also explores how lifestyle modifications in addition to surgical interventions may have a more dramatic impact than either intervention alone on overall patient outcomes. At four years, participants of a multimodal weight loss program lost 4.7% of the initial body weight compared to 1.1% with standard care alone; indicating that by addressing behavioral modifications, identifying barriers to weight loss, and providing accountability, multimodal approaches are more effective at sustaining weight loss [22].

In obese patients, as BMI increases, there is an associated decrease in GFR compared to normal-weight patients. The exact mechanism underlying GFR improvements following bariatric surgery is not well understood, but likely includes reductions in the pro-inflammatory state, changes in blood flow, and decreased renal sclerosis [23,24]. The mechanistic effects of lifestyle modifications prior to bariatric surgery in patients undergoing lifestyle interventions could not be explored within the current study due to its retrospective approach. However, future studies exploring the changes in renal perfusion and glomerular sclerosis may provide insight into the impact of lifestyle modifications prior to surgery with regards to long-term renal function following weight loss.

This study suggests that patients without hyperlipidemia at baseline have a greater improvement in GFR compared to patients with hyperlipidemia. This study revealed a 26% decrease in triglycerides one year after bariatric surgery, which is consistent with a previous study where a 41% decrease in triglycerides, 19% decrease in LDL levels, and 23% increase in HDL levels were observed [25]. Elevated triglycerides play a significant role in the development of arteriosclerosis, and individuals taking triglyceride-lowering therapy have a 16% risk reduction of major cardiovascular events. Previous studies have indicated that hyperlipidemia may contribute to renal injury through increased lipid deposition, resulting in oxidative stress and endothelial damage [26,27]. Therefore, patients without hyperlipidemia may have greater improvement in GFR due to a reduction of direct renal injury. Additionally, hyperlipidemia may cause focal glomerulosclerosis through direct podocyte toxicity [28]. Due to the retrospective nature of the study, the exact mechanisms for renal improvement in this population could not be determined and future studies with larger sample sizes and long-term follow-up are needed to determine the etiology of the noted improvements.

This study highlights the importance of lifestyle modifications prior to bariatric surgery as a treatment option for CKD patients with obesity. In specific populations, bariatric surgery can have beneficial outcomes, including increased blood sugar control, diabetes remission, and decreased comorbid medication usage [29]. However, bariatric surgery has the potential to cause severe complications such as infection, vascular injury, and nutritional deficiencies [30]. To minimize these risks, medication management and the optimization of lifestyle intervention prior to surgery can improve overall outcomes.

The primary limitation of the study was a small sample size (n = 55) with limited ethnicity distribution. This limits the generalizability of this study to larger, more diverse populations. Additionally, long-term follow-up was limited to only one-year post-intervention. Future studies exploring the long-term effects of lifestyle modifications prior to bariatric surgery and assessments of renal perfusion and glomerular sclerosis in this population will provide additional information about the potential mechanisms associated with improved renal function after lifestyle and surgical interventions.

## Conclusions

This study demonstrated that weight loss after bariatric surgery combined with lifestyle interventions is associated with significant improvements in renal function. These noted improvements indicate that a real-world, multimodal, lifestyle-modification weight loss program prior to bariatric surgery may play an important role in GFR improvement. Additionally, this study found that patients without hyperlipidemia had greater GFR improvement after bariatric surgery, indicating that patients with or without certain comorbidities may further benefit from lifestyle interventions prior to bariatric surgery. The findings of this study further support bariatric surgery as a potential treatment option for CKD patients with obesity to prevent disease progression and reduce cardiovascular risk factors.
